# Tumour of the Upper Jaw

**Published:** 1872-08

**Authors:** D. D. Bramble

**Affiliations:** Professor of Surgery in the Cincinnati College of Medicine and Surgery.


### ARTICLE V.

### Tumour of the Upper Jaw.

BY D. D. BRAMBLE, M. D.

Professor of Surgery in the Cincinnati College of Medicine and Surgery.

Under the title of tumours are comprised many diseases to which both sexes of all ages, and nearly every part of the body are liable. They possess many characters in common, yet at the same time they have important practical distinctions among themselves. These growths deviate from their parent structure, and contribute nothing to the performance of its function. Their mode of life is peculiar to themselves; they increase or diminish in size independently of the forces which govern the parts from which they deviate.

Tumours occur for the most part singly in the body, but sometimes are multiple. They are divided into two great classes, the innocent and the malignant. The one term expresses harmlessness of the tumour when left, and curability of it when excised ; the other the opposite of these propositions.

We meet with tumours of the upper jaw, serious in character and of a large size, involving great portions of the bone. Caution should be exercised not to mistake swellings of the upper jaw, inflammation and abscess of the antrum, for solid tumours. Unfortunately, the antrum is subject to malignant affections, occurring at all periods, both in young subjects and those advanced in life. The patient complains of an aching in the jaws, and of swelling, which, perhaps, is supposed to arise from a decayed tooth. The jaw swells out very rapidly, and then, after a short time, the nostril becomes obstructed, and there is discharge from it. The walls of the antrum are expanded, soft, and pulpy. Upon examination, we find a tumour filling up the whole cavity or the antrum.

The disease advances rapidly, and generally in a few weeks the tumour will have extended back to the throat, interfering with deglutition. The bones become wasted from pressure; patient gradually loses his health, emaciates, bowels are affected from swallowing the putrid discharge from the ulcerated surface of the tumour; and he has occasional hemorrhage which reduces him in strength still farther. These malignant tumours of the antrum go on rapidly, and the patient generally dies very miserably, from the discharge and hemorrhage, within half a year of the commencement of the disease. Fortunately all tumours of the upper jaw are not of this character; the bone and the periosteum become involved; the disease sometimes commencing from accident, as a stroke on the face with some hard substance; sometimes appearing spontaneously; that is, without any known cause.

These tumours are met with in all stages of their growth, and of various sizes. Their character is very different to the one described above. The tumour is limited, not involving all the neighboring parts, but goes on increasing very gradually. Growing year after year, at last it attains great bulk. The tumour feels hard and solid; the surface is very often lobulated; it is inconvenient from its size, yet it is not hurtful, and will not destroy the patient. The one tumour can scarcely be interfered with by a surgical operation with any propriety, while the other can be taken away safely and certainly.

The history of a tumour of the character just described, which has been in progress for several years, and has been removed once before, as follows:

Miss J—, aged 42, stout, healthy, and florid complexion, of a mixed sanguineous and lymphatic temperament. Can trace no hereditary predisposition to the complaint. Has always enjoyed good health. Dates the origin of the tumour four years back, and attributes it to no known cause; first observed a small lump at the lower border of the malar bone, and from that time the tumour gradually increased in size. In two years it attained the size of a quail egg. The family physician, on examination at this time, found the growth was very solid, similating exostosis, but as the case had made such slow progress, he advised non-interference. He saw the case three months later, when the tumour, without any apparent cause, had increased treble the size it was at the former examination. Whereupon he advised its removal, which was accordingly done, the wound healing in a reasonable time after contending with an attack of eresypelas; but it had not been well more than a week when it began to grow again, increasing in size very rapidly, so much so that in one year after, at the time we removed it, it weighed twelve ounces. The shape of the tumour was, at this time, irregular and slightly lobulated, about six inches long by three and a half inches in depth, occupying the whole side of the cheek, and extending under the lobe of the ear as well as over lapping it. Forwards it extended nearly to the angle of the mouth, and backwards about eight inches and a half. The integument was natural over the greater part of it, though the most prominent portion was rather reddish, and enlarged blood-vessels ran over its surface; there was a superficial ulceration at the center. To the sensation of touch it was firm as if fibrous.

It did not give the patient much pain, except when it was injudiciously handled. It caused a great deal of disfigurement from its bulk and position on the face. Considering it benign, taking all things into consideration, and with the patient in good health, we deemed it advisable to remove the tumour.

On the 16th of October, 1871, the patient being under the influence of chloroform, I made an incision across the transverse diameter of the tumour from below upwards; a second incision was made crossing the first at right angles at the center of the tumour, and the flaps were dissected back, laying bare the tumour, which I grasped and lifted from its bed, and quickly removed by a few strokes of the scalpel. The hemorrhage was restrained by pressure during the operation.

At the moment the tumour was broken loose from its bony attachment, respiration ceased, although no chloroform had been given for three minutes. For one and a half minutes there was no breathing, and life seemed nearly extinct for half an hour. We resorted to artificial respiration, applied heat and synapisms to the lower extremities, ammonia to nostrils, and, as soon as she could swallow, administered ammonia and brandy, and bathed her with camphor spirits.

It was one hour and a half before we could resume the operation, and then without the use of chloroform. Removed all of the facial portion of malar bone as well as the entire zygomatic arch; also molar process of superior maxilla, making a communication with the antrum of Highmore. I carefully dissected and removed every vestige of the growth. The tumour itself weighed twelve ounces.

The flaps were then brought together with isinglass plaster, aided by interrupted sutures. Water dressings were applied. There were no untoward symptoms during the healing process. Six weeks from date of operation the wound was healed and pronounced perfectly well.

Case II.—Fibrous tumour of upper jaw, of two years standing, in a young lady, aged 21, of nervous temperament. Healthy, never suffered from any disease. The rest of the family, of which there are five, are perfectly healthy, never having had any swellings or morbid growths about them. In this case, the first observable symptoms were looseness of teeth and soreness of gums. At the time of the operation, four teeth had fallen out, or rather they had been removed by the patient using her thumb and fingers for a tooth forceps.

The growth at this time had so increased in size on the anterior or external surface of alveolar process as to press against the cheek and make a very prominent projection on side of face. Downwards, in the space where the teeth had been removed, it had grown so as to prevent the closure of the teeth; hence, she was unable to masticate her food. On this account has been failing in health, in flesh and strength.

This growth I removed on the 24th of April, 1872, patient under the influence of chloroform. The operation consisted in removing the entire alveolar process between the first lateral incisior and last molar tooth, and a considerable portion of the body of superior maxilla, leaving in position the incisor and last molar. The diseased portion was removed through the mouth without making any external wound, by means of scalpel, chisel and bone forceps.

The after treatment consisted in washing the wound by means of a syringe; then saturating cotton in a weak solution of carbolic acid and introducing it into the cavity. The first 36 hours succeeding the operation there was much vomiting, the result of the blood swallowed. Soreness was considerable for several days, but little suppuration. The case progressed very favorably; at the present writing is perfectly well, only leaving a large space to be filled up by artificial means, so as to prevent a falling in of the cheek.—*Cincinnati Med. News.*

---

